# Prevalence of Fragile X syndrome in Georgian patients with autism spectrum disorder and/or intellectual disability: cross-sectional study and review of current approaches

**DOI:** 10.3389/fped.2025.1645386

**Published:** 2025-09-26

**Authors:** Nazi Tabatadze, Otar Koniashvili, Gia Melikishvili, Thierry Bienvenu, Laurence Cuisset, Flora Tassone, Dragana D. Protic, Nino Naneishvili, Maia Zarandia, Sopo Gverdtsiteli, Sophio Kakabadze, Tamar Gachechiladze, Mariam Melikishvili, Zurab Kuchukashvili, Tamaz Giunashvili, Ketevan Silagava, Giorgi Mamardashvili, Mariam Chipashvili, Ketevan Barabadze, Leonard Abbeduto, Randi J. Hagerman

**Affiliations:** ^1^ Faculty of Medicine, Ivane Javakhishvili Tbilisi State University, Tbilisi, Georgia; ^2^ Department of Pediatrics, MediClubGeorgia Medical Center, Tbilisi, Georgia; ^3^ Faculty of Medicine, Tbilisi State Medical University, Tbilisi, Georgia; ^4^ Université Paris Cité, Institute of Psychiatry and Neuroscience of Paris (IPNP), INSERM U1266, “Genetic vulnerability to addictive and psychiatric disorders” Team, Paris, France; ^5^ Service de médecine génomique des maladies de système et d’organe, Hôpital Cochin, Assistance Publique, Centre Université de Paris Cité, Paris, France; ^6^ Assistance Publique – Hôpitaux de Paris, APHP, Centre Universitaire Paris, Hôpital Cochin, Laboratoire de Génétique et Biologie Moléculaires, Paris, France; ^7^ Medical Investigation of Neurodevelopmental Disorders Institute, University of California Davis, Sacramento, CA, United States; ^8^ Department of Biochemistry and Molecular Medicine, School of Medicine, University of California Davis, Sacramento, CA, United States; ^9^ Department of Pharmacology, Clinical Pharmacology and Toxicology, Faculty of Medicine, University of Belgrade, Belgrade, Serbia; ^10^ Fragile X Clinic, Special Hospital for Cerebral Palsy and Developmental Neurology, Belgrade, Serbia; ^11^ Center for Mental Health and Prevention of Addiction, Tbilisi, Georgia; ^12^ Department of Molecular and Medical Genetics, Tbilisi State Medical University, Tbilisi, Georgia; ^13^ Department of Epilepsy Genetics and Personalized Medicine, Danish Epilepsy Center, Dianalund, Denmark; ^14^ Department of Regional Health Research, University of Southern Denmark, Odense, Denmark; ^15^ Department of Internal Medicine, Ascension Saint Joseph Hospital, Chicago, IL, United States; ^16^ Faculty of Exact and Natural Science, Ivane Javakhishvili Tbilisi State University, Tbilisi, Georgia; ^17^ Department of Pharmacology, Tbilisi State University, Tbilisi, Georgia; ^18^ Tbilisi State Medical University and High Technology Medical Center University Clinic, Tbilisi, Georgia; ^19^ MIND Institute, University of California Davis Health System, Sacramento, CA, United States; ^20^ Department of Psychiatry and Behavioral Sciences, University of California Davis School of Medicine, Sacramento, CA, United States; ^21^ Department of Pediatrics, School of Medicine, University of California, Sacramento, CA, United States

**Keywords:** Fragile X syndrome, *FMR1*, intellectual disability, autism spectrum disorder, prevalence, Georgia, genetic testing

## Abstract

Fragile X syndrome (FXS) is the most common inherited form of intellectual disability (ID) and autism spectrum disorder (ASD). Despite its clinical importance, data on FXS prevalence in Georgia remains limited. This study aims to assess the prevalence of FXS in individuals with ID and/or ASD in Georgia and to review current diagnostic and management approaches. A total of 441 patients (*n* = 332 males and *n* = 109 females) diagnosed with ID and/or ASD based on DSM-5 criteria underwent genetic testing for FXS using a PCR-based approach. The FXS full mutation was identified in 25 patients (5.7%), and four individuals were carriers of the premutation. One patient had a large FMR1 deletion, thus the prevalence of the full mutation (FM) was 5.9%, and the prevalence of a premutation was 0.9%. The FXS-positive cohort showed a significant male predominance (80.77%). Among patients with ASD, 1.9% tested positive for FXS, and these individuals displayed more severe behavioral problems, requiring more intensive intervention. Phenotypic features such as a long face (76.9%), joint hypermobility (61.5%), and flat feet (53.8%) were commonly observed. The study underscores a significant diagnostic delay, with the average age of clinical ID/ASD diagnosis at 8.42 years and a lag of 4.63 years before FXS is identified. Compared to the U.S., where FXS diagnosis occurs at 35–41 months, Georgia faces significant barriers, including low awareness, lack of early screening, and limited access to genetic testing. Efforts to address these challenges include public awareness campaigns and integration of early genetic testing protocols.

## 1 Introduction

Fragile X syndrome (FXS) is the most common inherited form of intellectual disability, with a prevalence of approximately 1 in 4,000 to 1 in 5,000 males and 1 in 6,000 to 1 in 8,000 females (1). The behavioral features of FXS are variable, but it is estimated to account for 1%–6% of autism spectrum disorders (ASD), making it the most common single-gene condition associated with ASD (2).

FXS is caused by a deficiency or absence of the Fragile X messenger ribonucleoprotein (FMRP), primarily due to the expansion of CGG trinucleotide repeats in the promoter region of the fragile X messenger ribonucleoprotein 1 (FMR1) gene, leading to hypermethylation and epigenetic silencing. Individuals with more than 200 CGG trinucleotide repeats have full mutation (FM) leading to FXS, whereas those with 55–200 repeats carry a premutation (PM) (3). In addition to CGG repeat expansions, other genetic changes such as point variations, complete or partial deletions, and splicing errors have also been reported to cause FXS (4, 5). Once diagnosed, tailored preventive care is warranted from infancy through early adulthood (6). This proactive approach helps to improve developmental outcomes and enhances the quality of life for individuals with FXS.

This report is the first to focus on the targeted population-based prevalence of FXS in Georgia. Population-based studies are essential to provide a comprehensive understanding of the true extent of the challenges associated with the condition, identify at-risk groups, and inform health policy and resource allocation.

## 2 Materials and methods

A total of 441 individual patients meeting the inclusion criteria for Intellectual and Developmental Disabilities (IDD) and/or ASD were selected from the two largest cities of Georgia, Tbilisi and Batumi. The diagnoses of IDD and/or ASD were established based on DSM-5 criteria (5th ed.; DSM-5; American Psychiatric Association, 2013) and further supported by ancillary assessments, including the Autism Diagnostic Observation Schedule (ADOS), Autism Diagnostic Interview-Revised (ADI-R), Wechsler Preschool and Primary Scale of Intelligence (WPPSI), Wechsler Intelligence Scale for Children (WISC), and Vineland Adaptive Behavior Scales, Third Edition (Vineland-3).

The first cohort of 116 patients was tested with the FastFraX™ *FMR1* Identification Kit (The Biofactory, Singapore), a PCR-based assay that uses melt curve analysis to detect CGG repeat expansions in the fragile X messenger ribonucleoprotein 1 (*FMR1*) gene. As an external quality control, all positive results were retested with triplet repeat primed PCR using AmplideX® *FMR1* PCR (Asuragen, Austin, TX) at the Genomic Medicine Laboratory, Hôpital Cochin, France. The second cohort of 325 patients was tested directly using the AmplideX® PCR/CE *FMR1* Kit (Asuragen, Austin, TX).

CGG repeats were categorized according to ACMG guidelines: Normal: <44 repeats (ACMG); Intermediate: 45–54 repeats (ACMG); Premutation: 55–200 repeats; Full mutation: >200 repeats (7).

In one patient, high clinical suspicion and an abnormal PCR amplification curve suggested a possible deletion or copy number variation, prompting us to perform array-CGH. The analysis was performed at the UC Davis MIND Institute, California. Copy number variants (CNVs) were compared with public databases, including DGV and DECIPHER, and classified according to established guidelines Additional family testing was performed in mother, two sisters and his maternal uncle after obtaining informed consent.

Clinical information was collected retrospectively from medical records. This included demographic details, clinical history, neurological, behavioral and developmental assessments, laboratory findings, imaging studies, and treatment history.

## 3 Results

A total of 441 patients were genetically evaluated for FXS, of whom 75.28% (*n* = 332) were male and 24.72% (*n* = 109) female. Among them, 59.88% (*n* = 264) were diagnosed with ASD, 89.83% (*n* = 396) had ID, and 19.77% (*n* = 87) had both ASD and ID. The mean age at molecular diagnosis of FXS was 13.05 years, compared to a mean age of 8.42 years at clinical diagnosis of ID or ASD.

PCR analysis detected FM in (*N* = 25) patients, while an additional (*N* = 4) patients had PM. Additionally, (*N* = 1) patient had a large deletion identified through Array-CGH, bringing the FM rate in this cohort to approximately 5.9% and 6.8%, respectively, (30/441) with any fragile X mutation.

The cohort had a significant male predominance, with 80.77% of the patients being male and 19.23% female, resulting in a sex ratio of approximately 4.2:1. Among the patients with the full mutation, 21 were male and 5 were female. All male patients with the full mutation had moderate to severe ID, and 4 were additionally diagnosed with both ID and ASD. Among the female patients, 4 were diagnosed with mild ID, and 1 with ASD. All four patients with the premutation exhibited learning disabilities, a feature consistently documented in previous studies (8).

Among the 26 patients with identified fragile X mutations, several phenotypic features were documented (Table 1). The most common was a long and narrow face (76.9%, *n* = 20), followed by joint hypermobility (61.5%, *n* = 16) and flat feet (53.9%, *n* = 14). Other frequent findings included prominent ears (50.0%, *n* = 13), soft and velvety skin (50.0%, *n* = 13), and a prominent jaw (38.5%, *n* = 10). Less frequent but notable features included constipation (34.6%, *n* = 9), sleep disorders (23.1%, *n* = 6), and seizures (15.4%, *n* = 4). Macroorchidism was present in all postpubertal males (100%, *n* = 4). Obesity was observed in 11.5% (*n* = 3), while macrocephaly and strabismus were each present in 7.7% (*n* = 2).

**Table 1:** Phenotypic features in patients with fragile X mutations.

Phenotypic feature	Percentage (%)	*N* (Count)
Long and narrow face	76.92	20
Joint hypermobility	61.54	16
Flat feet	53.85	14
Prominent ears	50.0	13
Soft and velvety skin	50.0	13
Prominent jaw	38.46	10
Constipation	34.62	9
Sleep disorders	23.08	6
Ear infections	19.23	5
Seizures	15.38	4
Macroorchidism (Postpubertal males)	100	4
Obesity	11.54	3
Macrocephaly	7.69	2
Strabismus	7.69	2

Beyond these physical manifestations, diagnostic subgrouping further clarified the clinical picture. Of the 441 patients, 264 (59.9%) were diagnosed with ASD. Within this group, 5 patients (1.9%) also tested positive for FXS (FXS-ASD), while the remaining 259 (98.1%) had ASD alone. Importantly, patients with FXS-ASD exhibited more severe behavioral difficulties—including anxiety, aggression, and self-injurious behaviors—than those with idiopathic ASD or FXS alone.

## 4 Discussion

Developing countries continue to struggle with the challenges associated with early diagnosis and treatment of FXS. This struggle is further compounded by the fact that FXS remains poorly understood among senior medical students in developing countries. One study found that none of the cohorts achieved a 60% passing threshold in FXS knowledge, with most students reporting inadequate education on neurodevelopmental disorders (NDDs). Barriers such as stigma, limited access to testing, the financial burden of testing, and systemic health care deficiencies exacerbate delayed diagnoses and poor care (9).

In the US, boys with FXS are typically diagnosed with FXS at an average age of 35–37 months, while girls are diagnosed later, at around 41.6 months. There is an average delay of 24 months between the first concern (11.6 months for boys, 16 months for girls) and formal diagnosis of FXS (10). In contrast, based on our study in the Georgian cohort, the mean age at molecular diagnosis of FXS was 13.05 years, compared to a mean age of 8.42 years at clinical diagnosis of ID or ASD, reflecting an average diagnostic delay of 4.63 years.

To address the delay in diagnosis and the associated challenges, we have conducted numerous awareness campaigns and established active social platforms. These efforts have significantly increased Georgia's position on the global map for FXS awareness and advocacy. This proactive approach aims to improve developmental outcomes and enhance the quality of life for individuals with FXS. While the American Academy of Pediatrics (AAP) recommends testing patients with ASD and/or ID for FXS, limited resources and awareness in most developing countries result in a two-step diagnostic process. This involves first identifying physical stigmata associated with FXS, followed by genetic testing. Such an approach risks overemphasizing physical features, potentially delaying early interventions that are critical to improving psychosocial outcomes.

In addition, the lack of resources often makes multidisciplinary care inaccessible for patients with FXS, further limiting their access to comprehensive treatment.

The current practice in the management of FXS involves both non-pharmacologic and pharmacologic approaches. Although the evidence base for the efficacy of a specific treatment approach is quite limited. Non-pharmacological interventions, such as physiotherapy, occupational therapy, and speech and language therapy, are essential in addressing motor and communication delays. Applied Behavior Analysis (ABA) is particularly beneficial for children with FXS, especially those with comorbid ASD, to improve social and communication deficits. Cognitive-behavioral therapy (CBT) is effective in managing anxiety, attention-deficit hyperactivity disorder (ADHD), and social deficits in higher-functioning individuals (11, 12). Educational programs that emphasize sensory integration, social interaction, and life skills contribute significantly to improvements in adaptive behavior. Pharmacologically, targeted treatments aim to address the dysregulated pathways characteristic of FXS. Medications such as selective serotonin reuptake inhibitors (SSRIs), psychostimulants, antiepileptics such as lamotrigine, and antipsychotics are used to manage symptoms such as anxiety, ADHD, seizures, and behavioral problems. Novel treatments, including metformin and cannabidiol (CBD), have shown promise in modulating synaptic plasticity and improving behavioral outcomes by targeting molecular pathways such as mTOR and the endocannabinoid system (13–16).

Over the past decade, considerable research has been conducted to better understand the pathophysiology of FXS and to develop targeted treatments. While some approaches have shown promise, the results have been mixed. For example, metabotropic glutamate receptor 5 (mGluR5) antagonists, such as mavoglurant, failed to show significant improvements in behavioral symptoms compared to placebo (17). Similarly, GABAergic system modulators such as arbaclofen showed some improvement but lacked statistical significance (18). In contrast, Phosphodiesterase-4D (PDE4D) inhibitors, such as zatolmilast, have shown encouraging results, including improvements in cognitive function and behavior, highlighting their promise as a therapeutic option for FXS (19).

Advances in gene therapy have also shown potential. Recent studies have shown that targeting specific R-loops can induce the contraction of CGG repeats and reactivate the silenced *FMR1* gene in FXS cells, offering a novel and promising approach (20).

## 5 Conclusion

In conclusion, FXS remains a significant yet underdiagnosed cause of ID and ASD, particularly in resource-limited settings. Georgia continues to face major challenges in autism and FXS care, particularly due to the lack of standardized referral pathways after a clinical diagnosis and the limited availability of resources for patient education and advocacy. Notably, in our cohort the male-to-female prevalence ratio of FXS was 4.2:1, compared with the approximately 2:1 ratio reported internationally. This disparity likely reflects the historical under-recognition of FXS in females, as the condition has often been perceived primarily as a disorder affecting boys with ID and ASD. Consequently, many affected girls may remain undiagnosed, underscoring the importance of raising awareness of the female phenotype and ensuring more inclusive diagnostic approaches. To address gaps, in close collaboration with NFXF we have established a Fragile X clinic and national patient registry, also launched an annual Fragile X Awareness campaign. As a next step, we are preparing a mixed-methods study that will integrate qualitative and quantitative approaches to evaluate current knowledge and practices among healthcare professionals. Together, these initiatives aim to enhance early identification, strengthen patient and family support systems, and promote nationwide advocacy for individuals affected by FXS and related conditions. Our study underscores the urgent need to integrate early genetic testing into routine pediatric and neurodevelopmental care pathways, as this would facilitate earlier diagnosis and enable tailored management strategies that ultimately improve patient outcomes. Increased public and professional education about FXS, combined with global collaboration, holds promise for reducing diagnostic delays, improving access to multidisciplinary care, and ultimately improving the quality of life for individuals affected by this condition.

## Data Availability

The original contributions presented in the study are included in the article/Supplementary Material, further inquiries can be directed to the corresponding author.
